# MSMEG_3955 from *Mycobacterium smegmatis* is a FMN bounded homotrimeric NAD(P)H:Flavin mononucleotide (FMN) oxidoreductase

**DOI:** 10.1186/s12866-021-02330-y

**Published:** 2021-11-19

**Authors:** Neha Khosla, Seema Madhumal Thayil, Rajinder Kaur, Anup Kumar Kesavan

**Affiliations:** 1 Department of Molecular Biology and Biochemistry, Guru Nanak Dev University, Amritsar, Punjab 143005 India; 2 Department of Botanical and Environmental Sciences, Guru Nanak Dev University, Amritsar, Punjab 143005 India; 3 Current Address: Department of Biotechnology & Microbiology, Kannur University, Dr. E.K. Janaki Ammal Campus, Palayad, Thalassery, Kannur, Kerala India

## Abstract

**Background:**

Tuberculosis (TB) remains an important public health problem since it is the major cause of elevated morbidity and mortality globally. Previous works have shown that *Mycobacterium tuberculosis (Mtb)*; the prime causative agent of the deadly disease has dormancy survival regulator (DosR) regulon**,** a two-component regulatory system which controls the transcription of more than 50 genes. However, the structure and detailed functions of these DosR regulated genes are largely undetermined. Out of many DosR regulon genes, *Rv3131* gets up regulated in hypoxic conditions and was believed to encode for a nitroreductase flavoprotein. The utilization of mycobacteria-specific model systems has greatly added to our understanding of the molecular mechanisms involved in the life cycle and pathogenesis of *Mtb.*

**Results:**

In this study the non-pathogenic mycobacterial model organism *M*ycobacterium *smegmatis* (*Msmeg*) was used to reveal the structure and function of MSMEG_3955*;* which is a homologue of Rv3131 from *Mtb*. Using chromatography and spectroscopy techniques it was revealed that cofactor flavin mononucleotide (FMN) was bound to flavoprotein MSMEG_3955. Consistent with the homology modelling predictions, Circular Dichroism (CD) analysis indicated that the MSMEG_3955 is composed of 39.3% α-helix and 24.9% β-pleated sheets. In contrast to the current notions, the enzymatic assays performed in the present study revealed that MSMEG_3955 was not capable of reducing nitro substrates but showed NADPH dependent FMN oxidoreductase activity. Also, gel permeation chromatography, dynamic light scattering and native acidic gels showed that MSMEG_3955 exists as a homotrimer. Furthermore, the presence of NADPH dependent FMN oxidoreductase and homotrimeric existence could be an alternative function of the protein to help the bacteria survive in dormant state or may be involved in other biochemical pathways.

**Conclusion:**

MSMEG_3955 is a FMN bound flavoprotein, which exits as a trimer under in vitro conditions. There is no disulphide linkages in between the three protomers of the homotrimer MSMEG_3955. It has a NADPH dependent FMN oxidoreductase activity.

**Supplementary Information:**

The online version contains supplementary material available at 10.1186/s12866-021-02330-y.

## Introduction

*Mtb* can reside within host tissues for years or even decades without causing the disease, the mechanism known as latency or dormancy [1]. The sensitivity of dormant bacterium against antibiotics is far less than the active bacterium, which requires long course of antibiotics [2]. One third of the population has latent TB, placing them on risk of active TB in future [3]. Thus to fight against TB, understanding the genes involved in latency are of critical interest. *Mtb* encodes a set of 50 genes called the dormancy survival regulator (DosR) regulon that are expressed during stress, hypoxia, NO stress and nutrient starvation and helps the bacteria to survive in latency [4–6]. Genes within the DosR regulon are involved in multiple processes i.e., central metabolism, energy generation and gene regulation. However, majority of them are not functionally characterized. The gene *Rv3131* is a part of DosR regulon and it belongs to the *acr co-regulated gene* (*Acg*). *Acg* gene is known to be essential for growth and virulence in vivo [6]. *Rv3131* gene product is also required for mycobacterial growth which has been studied using high density mutagenesis [4, 7–9]. It is considered to be a putative nitroreductase flavoprotein. Flavins are extremely versatile cofactors that undergo redox reaction by accepting either one electron or two electrons, alternating between the oxidized and reduced states. Flavoenzymes are the class of oxidizing enzymes containing an electron acceptor FMN or Flavin adenine dinucleotide (FAD). These electron carriers accommodate 90% of apoprotein through non-covalent interactions [10]. Flavin reductases are the enzymes which reduce free flavins (FMN, FAD/riboflavin) through reduced pyridine nucleotide, NADH or NADPH [11]. Dihydroflavins produced thus participate in a variety of biological prokaryotic redox reactions include hydroxylation of phenolic compounds, epoxidation of styrene, reduction and mobilization of iron from ferric complexes of siderophores etc. [12–14]. It is believed that the putative nitroreductase Rv3131 involve in detoxification of nitrogen- containing bi-products in the host, but the structure and functional role of Rv3131 in mycobacterial species is still unknown [3].

In the present study, the *Msmeg* is used as model organism to study the gene *MSMEG_3955* which is the homologue of gene *Rv3131* from *Mtb.* The gene *Rv3131* shares 63% identity to the *MSMEG_3955.* The characteristics of slow growing *Mtb* and fast growing *Msmeg* are quite similar, thus the use of the non-pathogenic and fast growing species as a model organism to study the virulence of *Mtb* is important [15]. The gene MSMEG_3955 was cloned and expressed to get its structural and functional insights.

## Experimental procedures

### Strains, reagents, plasmids, media and growth conditions

*M. smegmatis mc* ^*2*^ *155* was grown in Middlebrook 7H9 broth (HiMedia), containing 0.5% (v/v) glycerol and 0.05% Tween 80 and appropriate amount of Oleic acid dextrose catalase (OADC) (HiMedia) for 48 h aerobically at 37 °C. *E.coli* (DH5α) was used for DNA amplification and *E.coli* (BL21) (DE3) for overexpression of recombinant proteins. Both the strains were grown in LB broth (luria-bertani) (HiMedia) for 16 h, keeping the media in shaker with constant agitation at 180 rpm at 37 °C. The vector pET-28a was used for the over-expression of the recombinant protein MSMEG_3955. Kanamycin 50 μg/mL was routinely used in LB media during the growth of strains having vector pET-28a. Isopropyl β-D-1-thiogalactopyranoside (IPTG) (HiMedia) was used to induce the recombinant protein MSMEG_3955. Nickle nitrilotriacetate (Ni-NTA) agarose beads (Qiagen) were used for the purification of the expressed protein. Polymer chain reaction (PCR) products were gel purified through GEL/PCR DNA Fragments Extraction kit (IBI SCIENTIFIC, USA), and plasmid were isolated through High-Speed Plasmid Mini kit (IBI SCIENTIFIC, USA). Amicon Centrifugal Filter Unit (EMD Millipore) were used to concentrate and dialysed the proteins. Nitrofurantoin was purchased from MP Biomedicals. FMN, NADH and NADPH were from HiMedia. Urea, Sodium dodecyl sulfate (SDS) and Trichloroacetic acid (TCA) were obtained from HiMedia.

### Cloning and expression of recombinant protein

Genomic DNA was isolated from *Msmeg* using the hexadecyltrimethyl ammonium bromide (CTAB) method [16]. Spectrophotometrical analysis was performed to quantify the isolated genomic DNA. For the amplification of *MSMEG_3955,* forward primer and reverse primer were designed. The primer set used; forward, 5’CGCGGATCCATGAATACGCACTTCCCGGAT3’, reverse, 5’CCCAAGCTTCTACTCCGATTCCGGCTCGAA3’ (underline are Bam HI and Hind III restriction sites respectively). PCR was done with Phusion High Fidelity DNA Polymerase (NEB) in the Agilent thermocycler (USA) using following conditions for amplification; hot start at 98 °C for 3 min, denaturation at 98 °C for 30 s, annealing at 66 °C for 30 s, elongation at 72 °C for 45 s (35 cycles), followed by final elongation at 72 °C for 10 min. The amplified gene *MSMEG_3955* was cloned in the expression vector pET-28a and transformed in *E. coli* (DH5α) for amplification of recombinant clones. The positive clone was confirmed with restriction double digestion. The confirmed clone was further transformed in *E.coli* (BL21) (DE3) for the over-expression of recombinant protein MSMEG_3955. For the over-expression, the cells were grown in 3 mL of LB broth for overnight (16 h incubation) at 37 °C and subcultured to the fresh 500 mL LB broth. The subculture allowed to grow at 37 °C till the OD_600_ reach 0.4–0.6. For the induction of recombinant protein 1 mM IPTG was added to the culture after required OD_600_, keeping the culture at 25 °C for 24 h. All the cultures were kept in the shaker at optimum temperatures with constant shaking at 180 rpm. The cells were harvested by centrifugation and suspended in lysis buffer containing 50 mM Tris-HCl (pH -8.0), 500 mM NaCl, 100 μg/mL Lysozyme, 1 mM PMSF and 10 mM Imidazole. One gram pellet of cells were suspended in 1 mL of lysis buffer containing 50 mM Tris-HCl (pH -8.0), 500 mM NaCl, 10 mM imidazole, 100 μg/mL lysozyme and 0.02% NaN_3_. The cells were lysed using SONICS vibra cell sonicator at 37% amplitude with 5 s ON and 5 s OFF for 1–2 min (for each sample) or until the sample got clear. The samples were kept in ice during the sonication to avoid denaturation of protein during extraction as the sample was getting hot when sonication proceed. The sonicated cell lysates was centrifuged at max speed for 30 min. Supernatant containing recombinant protein MSMEG_3955 was collected in fresh tubes.

### Purification of recombinant protein

For the purification of recombinant protein, Ni^2+^-NTA agarose Quick-Star (Qiagen) was used. One milliliter of Ni-NTA slurry (0.5 mL bed volume) was added in the column and washed thrice with lysis buffer. After washing, 4 mL of clear cell lysate was added to the slurry in the column and mixed gently by shaking at 200 rpm at 4 °C for 1 h to get the bound protein. The flow-through was collected by removing bottom cap of the column. The bound protein was washed thrice with 5 bed volume (2.5 mL) wash buffer containing 50 mM Tris-HCl (pH -8.0), 300 mM NaCl, 40 mM imidazole and 0.02% NaN_3_. The washed fractions were collected for 10% SDS-PAGE analysis. The purified protein MSMEG_3955 was eluted 5 times with 0.5 mL of elution buffer containing 50 mM Tris-HCl (pH -8.0), 300 mM NaCl, 300 mM imidazole and 0.02% NaN_3_. The eluted protein was dialysed and concentrated using Amicon Centrifugal Filter Unit (EMD Millipore). Quantification of protein was done by Bradford method using bovine serum albumin (BSA) as standard [17].

### Estimation of reduced and non-reduced protein

SDS-PAGE was carried out to analyse the size of reduced and non-reduced protein MSMEG_3955. To determine the reduced form, the protein was treated with reducing buffer containing 12% SDS, 30% glycerol, 0.05% coomassie blue dye, 150 mM Tris-Hcl (pH -7.0) and 6% β-mercaptoethanol and run on 12% acidic SDS-PAGE. To determine the non-reduced form of recombinant protein, it was treated with non-reducing buffer containing 12% SDS, 30% glycerol, 0.05% coomassie blue dye, 150 mM Tris-Hcl (pH -7.0) and run on 12% acidic SDS-PAGE.

### Estimation of oligomeric state of protein

#### NATIVE-PAGE analysis

The oligomeric state of purified native protein was estimated by NATIVE-PAGE analysis. In this, 10 μg of the protein sample was loaded directly to the 12% NATIVE-PAGE, without any treatment. The loading buffer contains a coloured dye bromophenol blue and a density agent glycerol only.

#### Size exclusion chromatography

Oligomeric state of recombinant protein MSMEG_3955 was confirmed by gel permeation chromatography (GPC) using NGC Chromatography system on Enrich SEC 70 10/300 column (Bio-Rad) pre-equilibrated with wash buffer containing 50 mM NaH_2_PO_4_, 150 mM NaCl, 0.02% NaN_3_ (pH -8.0)_**.**_ The retention volume of MSMEG_3955 was compared with the standards BSA (132 kDa) and Lysozyme (14 kDa). The flow rate was maintained at 0.5 mL/min.

#### Dynamic light scattering (DLS)

Oligomeric state and purity of protein MSMEG_3955 was determined by peak analysis and overall polar dispersity using DLS. Purified 20 μM protein sample was analysed in disposable sizing cuvette using a path length of 1 cm at 20 °C in Malvern Zetasizer instrument.

### Homology modelling

The homology model of MSMEG_3955 was generated to explore its structural similarity with the conformation of MSMEG_3955 from CD. MSMEG_3955 shows 35% identity to protein MSMEG_5246, therefore it was used as a template to generate the 3 dimensional structure of MSMEG_3955. The FASTA format of amino acid sequence of protein was retrieved from Mycobrowser (http://mycobrowser.epfl.ch) for structure prediction. BLAST2 was used to obtain homologous entries of protein MSMEG_3955 from Protein Data Bank in NCBI for template search. Top hit template MSMEG_ 5246 PDB ID: 2ymv was used for homology modelling using SWISS-MODEL [18] a fully automated protein structure homology-modelling server (https://swissmodel.expasy.org/interactive). Generated homology model of protein MSMEG_3955 was monomer as template protein MSMEG_5246 used was monomer [19] PyMOL program was used to visualize the generated protein models [20]. The quality of generated protein was validated with quality factors such as RAMACHANDRAN PLOT as PROCHECK [21] (http://www.ebi.ac.uk/thornton-srv/software/PROCHECK/) to evaluate backbone and side chains of protein, Verify3D [22] (http://services.mbi.ucla.edu/Verify_3D/) was performed to measure the compatibility of the protein’s structure with its respective sequence and ERRAT [23] (http://services.mbi.ucla.edu/ERRAT/) was performed to find out overall reliability of generated 3D structure of protein.

### Prediction of secondary structure

The secondary structure of protein MSMEG_3955 was predicted by CD spectroscopy. The spectra provides an index of structure. The scanning measurements were made from 200 nm to 260 nm wavelength, with JASCO J-1500 Circular Dichroism spectrometer. MSMEG_3955 protein concentration used was 8 μM in 10 mM HEPES using a 0.1 cm path length quartz cuvette at 25 °C. The solvent spectrum was subtracted from the sample spectrum.

### Determination of cofactor

#### Thin layer chromatography (TLC)

TLC was performed to analyse the cofactor bound to the recombinant protein. The purified protein was denatured at 70 °C for 20 min. The samples were centrifuged at maximum speed for 20 min. The supernatant was spotted on thin layer of silica gel along with 5 μM of FMN, Riboflavin and FAD as control. The mobile phase used was 1-butanol-acetic acid-water (4:1:1, by volume) and the developed plate was visualized under UV light [24, 25].

#### UV-visible spectroscopy

The spectrophotometric confirmation of co-factor bound to the protein MSMEG_3955 was analysed using SDS treatment of holoprotein (Protein + cofactor) [26, 27]. UV-Visible spectra were recorded between 400 to 500 nm. The absorbance spectrum of the 100 μM protein in 10 mM Tris-HCl buffer, pH -8.0 was recorded. Twenty microliter fresh 10% SDS solution was added to the cuvette containing protein. After mixing, spectra were recorded every 5 min until no further changes were observed.

### Site directed mutagenesis (SDM)

Mutant was generated using PCR-based SDM described by Hoa et.al [28]. There are three cysteine residues present at three different sites 63, 70, 263 in the amino acid sequence of MSMEG_3955. Plasmid pET-28a containing required gene *MSMEG_3955* was used as a template for PCR. The amino acid cysteine (TGC) at position 63 was replaced with alanine (GCC). Phusion High Fidelity DNA Polymerase (NEB) was used in the Agilent thermocycler (USA). Two step PCR was performed using set of primer forward, 5’GATCTGCTCCTCAGCGCCGGCGCGGCGCTGCAC3’ and reverse, 5’GTGCAGCGCCGCGCCGCCGCTGAGGAGCAGATC3’. The following conditions were followed for amplification; hot start at 98 °C for 1 min, denaturation at 98 °C for 15 s, annealing at 72 °C for 3 min, elongation at 72 °C for 10 min (30 cycles). The amplified cDNA was treated with DpnI to digest methylated parental DNA and the newly synthesised cDNA was transformed in *E. coli* (DH5α) for amplification of the product of mutagenesis. The isolated positive clones were confirmed with sequencing and then transformed in *E. coli (BL21) (DE3)* for its overexpression.

### FMN reductase assay

#### UV-visible spectroscopy

Flavin reductases activities were carried out as described by Rakhi et al. with modification [29]. In the modified protocol, 2 mL of reaction volume with increased concentration of electron donor in Tris-HCl buffer was used. Two reaction mixtures were setup; for both the reactions, NADPH was used as an electron donor. For each reaction, the oxidation of electron donor was observed by decrease in the absorbance at 340 nm at 25 °C under aerobic conditions. In one experiment, FMN (50 μM), NADPH (200 μM) with 1.6 μM of enzyme/protein MSMEG_3955 in 50 mM Tris-HCl pH -7.6 in 2 mL reaction volume was used. NADPH was added just before placing the tube in spectrophotometer (Systronics double beam spectrophotometer 2202). In another experiment, NADPH (100 μM) with 1.6 μM of enzyme/protein MSMEG_3955 in 50 mM Tris-HCl pH -7.6 in 1 mL reaction volume was used keeping the reaction at 25 °C for 48 h. A control experiment was performed without enzyme. Both the experiments were performed in triplicates.

#### ^1^H NMR

The NADPH dependent FMN reductase activity of protein MSMEG_3955 was confirmed with ^1^H NMR spectroscopy. The 2 mL volume of reaction mixture was set that contain FMN (500 μM), NADPH (15 mM) with 30 μM of enzyme/protein MSMEG_3955 as catalyst in 50 mM Tris-HCl pH -7.6. The ^1^H NMR spectrum of FMN after 2 h incubation at 25 °C was recorded and compared with its oxidized form.

## Results

### Cloning, expression of protein

**Fig. 1:**
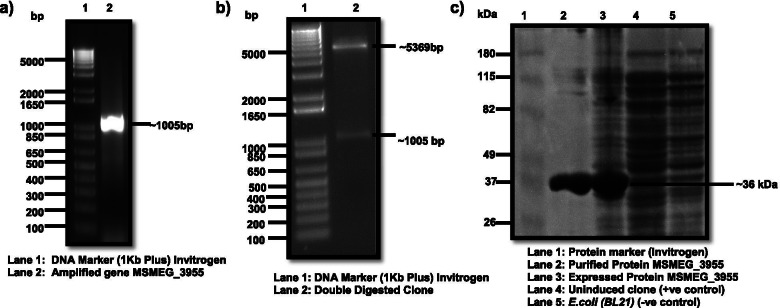
Electrophoretic gel pictures. **a** Gel picture of amplified gene *MSMEG_3955* from genomic DNA**. b** Gel picture of double digested clone of pET 28a vector containing gene *MSMEG_3955*. **c** Gel picture of 12% acidic SDS-PAGE of expressed reduced purified recombinant protein, expressed reduced crude protein along with controls

**Fig. 2:**
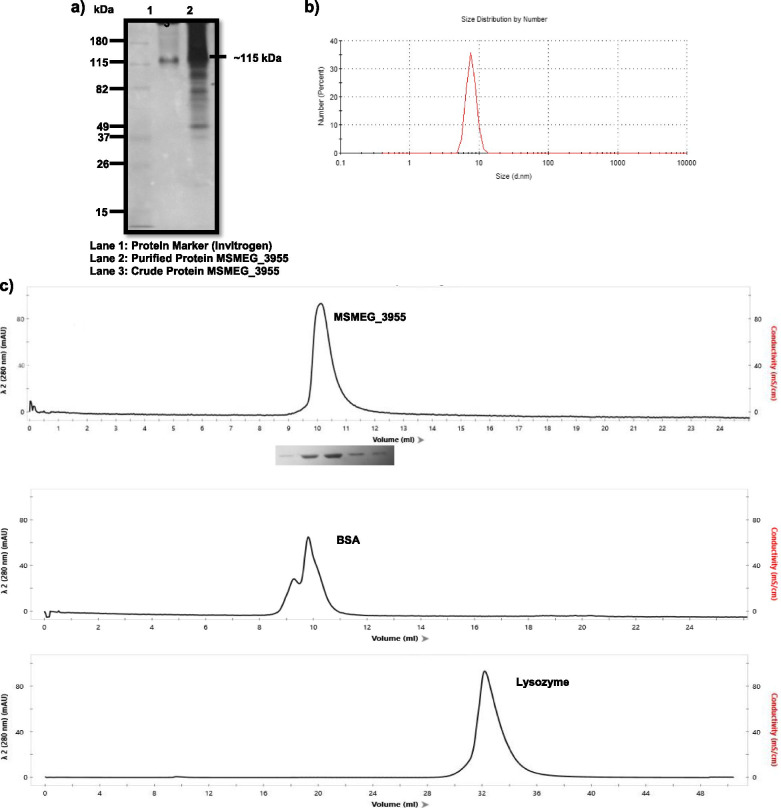
Polyacrylamide Gel Electrophoresis for analysis of the presence or absence of disulphide linkage within the homotrimeric protein. **a** Gel picture of wild-type and cys-63 mutated native protein MSMEG_3955 run on 12% NATIVE-PAGE. **b** Non-reduced protein MSMEG_3955 run on 12% SDS-PAGE

The gene *MSMEG_3955* shares 63% identity to the gene *Rv3131* in *Mtb.* (Both the sequences were aligned using BLASTP as shown in Fig. S1). The gene specific primers for PCR resulted ~ 1005 bp amplicon (Fig. 1a). The gel purified PCR product was cloned into the expression vector pET28a and confirmed by restriction double digestion (Fig. 1b) and DNA sequencing. The recombinant reduced protein was identified by SDS-PAGE with an expected protein size of ~ 36 kDa (Fig. 1c). The non-reduced protein gave the similar size of band in 12% acidic SDS-PAGE (Fig. 2b).

### Protein MSMEG_3955 is homotrimeric

**Fig. 3:**
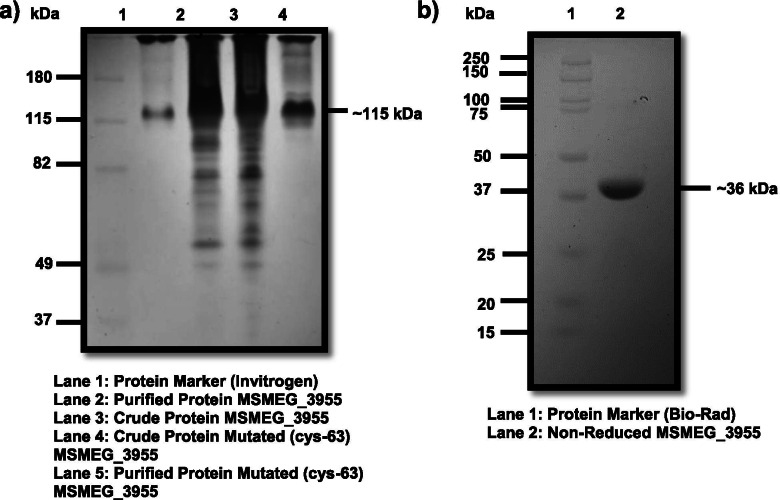
Oligomeric state analysis of protein MSMEG_3955**. a** Gel picture of native protein run on 10% NATIVE-PAGE. **b** Percentage particle size distribution graph of protein showing diameter of molecule ~ 7.8 nm, analysed by DLS. **c** Gel permeation chromatography showing elution profile of protein (~ 115 kDa) with BSA (132 kDa) and Lysozyme (14 kDa) as standards

The oligomeric state of protein is homotrimeric, since on NATIVE- PAGE the band of 10 μg protein corresponding to ~ 115 kDa was seen (Fig. 3a). This was also confirmed by performing size exclusion chromatography of the given protein. In order to compare NATIVE size of protein obtained from acidic NATIVE-PAGE electrophoresis, size exclusion chromatography was performed (Fig. 3c). Consideration and comparison of the size exclusion elution profile of MSMEG_3955 and the results of SDS-PAGE and NATIVE-PAGE suggests that the protein is an oligomer and it exists as homotrimer in the solution. Dynamic light scattering measurements exhibits sharp monodisperse peak of 20 μM protein centred around 8.7 diameter (nm) with molecular weight calculated at 105.6 ± 12.2 kDa [30–32], which also suggests the trimeric existence of MSMEG_3955 (Fig. 3b).

### Site directed mutagenesis

One of the cysteine residue was mutated at position 63 with alanine to see the change in oligomeric structure. Sanger sequencing of amplified mutated clone confirmed replacement of cysteine to alanine in the gene of *MSMEG_3955* at amino acid position 63. Nucleotide BLAST was performed between mutated gene sequence and wild type gene sequence of *MSMEG_3955* to confirm the mutation. The mutated protein was overexpressed and purified by Ni-NTA method. To determine any oligomeric alterations in the mutated native protein, 20 μg of mutated protein and 20 μg of wild type protein were run on NATIVE-PAGE. On SDS-PAGE (data not shown) and NATIVE-PAGE, the wild-type protein and the mutant protein had identical sizes (Fig. 2a).

### Homology modelling and CD analysis

**Fig. 4:**
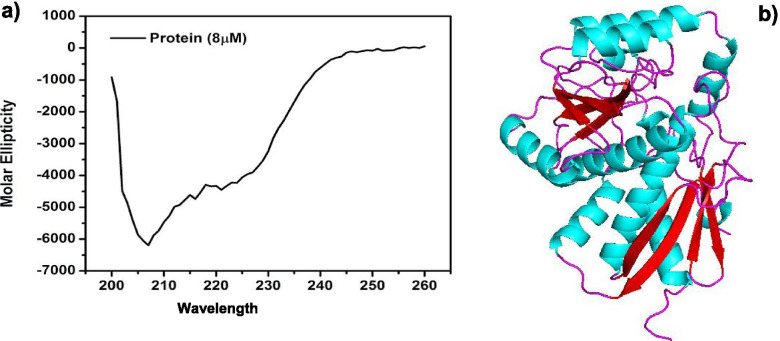
Secondary structure analysis of MSMEG_3955**. a** Graphical representation of Circular Dichroism spectrum of MSMEG_3955 protein (8 μM) in 10 mM HEPES. **b** Modelled structure of MSMEG_3955, Helices are colored blue, sheets are red and coils are purple

Three dimensional structure of monomeric MSMEG_3955 was obtained from homology modelling. The generated model MSMEG_3955 composed of 40% α-helix and 22% β-pleated sheets analysed using STRIDE programme [33]. The quality of monomeric protein was validated with PROCHECK, Verify 3D and ERRAT to assess their acceptability and were found suitable for structural analysis as seen in Fig. S4(a), S4(b), S4(C) resp. CD spectroscopy was performed to validate experimentally the secondary structural conformation of protein MSMEG_3955 predicted by homology modelling. Analysis of CD spectra suggested that MSMEG_3955 is composed of 39.3% α-helix, 24.9% β-pleated sheets, 19.1% turn and remaining part assumed to be randomly coiled (Fig. 4a). The CD spectra was analysed as described by Yang’s et.al [34]. These results are in close agreement with MSMEG_3955 homology model prediction for α-helix and β-pleated sheets contents (Fig. 4b).

### Cofactor FMN is bound to MSMEG_3955

**Fig. 5:**
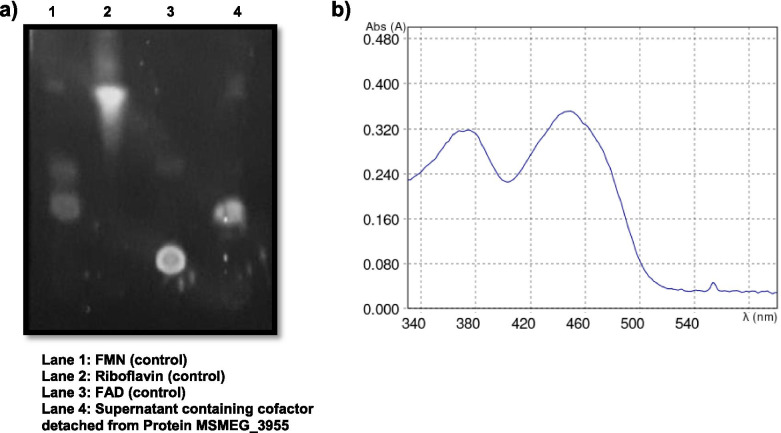
Analysis of cofactor bound to the protein MSMEG_3955. **a** The cofactor separated from protein MSMEG_3955 migrates along with FMN (control Lane-1) on thin layer chromatography (TLC). **b** UV Spectrum showing maximum absorbance at 446 nm wavelength for cofactor detached from protein treated with 10% SDS

The absorption spectra recorded using UV-Visible spectroscopy gave the peak at 446 nm after 10 min of SDS treatment to the protein MSMEG_3955. Since the FMN absorbs at 447 nm, UV-visible spectroscopy identified the cofactor bound to the protein MSMEG_3955 is FMN (Fig. 5a). The cofactor extracted by thermal denaturation of protein MSMEG_3955 co-migrated with FMN (control) on the silica gel, when kept in the mobile phase confirmed the cofactor FMN is bound to the protein MSMEG_3955. The chromatogram was seen under UV torch (Fig. 5b).

### MSMEG_3955 is a FMN reductase

**Fig. 6:**
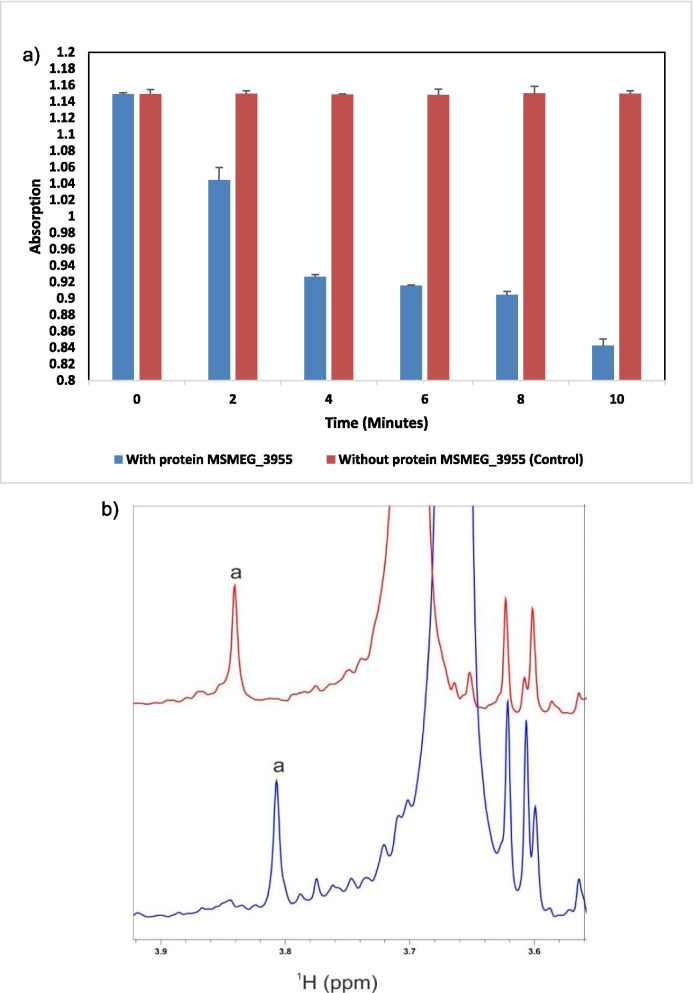
Measurement of FMN reductase activity using NADPH as an electron donor**. a** The protein/enzyme MSMEG_3955 showed oxidative NADPH dependent FMN reductase activity in contrast to control. **b** The selected region of ^1^H NMR spectra of FMN in oxidized (red) and reduced (blue) form recorded at 500 MHz, pH -7.6. The Flavin ring proton is represented by letter “**a**”

The MSMEG_3955 protein/enzyme showed oxidative NADPH dependent FMN reductase activity as oxidation of NADPH was seen. The decrease in NADPH absorbance seen in first experiment showed the enzyme MSMEG_3955 is reducing FMN provided. Readings were taken after addition of NADPH every 2 min interval for 10 min Fig. 6a). The absorbance of NADPH was zero which shows the whole FMN bound to the protein was reduced. Fig. S5 is a plot of absorbance vs time between 0 and 600 s for a protein in the first experiment. By Beer’s law, the absorbance of the solution is directly proportional to the concentration of the protein in the solution, so observing the absorbance as a function of time is basically the same as observing the concentration versus time.

The curve represent data fits a first-order reaction and straight fitting of plot ln[A] versus time will yield rate constant *(k) = − 2 × 10*^*− 3*^ *s*^*− 1*^*.* In the second set of experiment, NADPH absorbance was decreased as FMN bound to the enzyme itself was reduced. In the ^1^H NMR spectrum of FMN all the peaks were observed as reported in BMRB data for FMN (Entry number- bmse000257) shown in Fig. S2. The Flavin ring proton exhibit upfield chemical shift on reduction as reported earlier [35] labelled as “a” (Fig. 6b) and full spectra were shown in Fig. S3.

## Discussion

The latency mechanism is found to be associated with the dormancy survival regulator transcription factors which together constitute the DosR regulon [4, 36]. There are multiple genes present in the DosR regulon but the structure and function of most of their encoded proteins are unknown. Also, the DosR regulon associated proteins such as Acg family has evolved from the nitroreductase homodimer by gene duplication and fusion and then loss of one of the two active sites. This has resulted in a monomeric protein with a single active site but with an overall fold resembling the nitroreductase homodimer [19]. Therefore, it is believed that most of the DosR regulon encoded proteins may have the potential nitroreductase activity. In the present study we characterised a novel DosR regulon gene *MSMEG_3955* which was of *Msmeg* origin. MSMEG_3955 was found to be a homotrimeric and showed FMN reductase activity. Flavin biosynthesis is important for the action of *Mtb* as it lacks the flavin uptake mechanism [37]. Riboflavin biosynthesis enzymes have been found to be conserved in *Mycobacterium leprae*, regarded as a minimal mycobacterial genome [38, 39]. However, regulation of flavin synthesis in *Mtb* is poorly explored. More than 3% of *Mtb* genes are flavoproteins and are particularly rich in FAD-dependent acyl-CoA dehydrogenases required for lipid degradation [40, 41]. The present study provides the first impression of FMN related activity of a DosR regulon encoded protein. The results so obtained can add to our understanding of the physiological state of the *Mtb* during latency and could be of utmost interest in the fight against tuberculosis. In the present study it is observed that the reduced and non-reduced protein gave the similar size of bands in SDS-PAGE of ~ 36 kDa. On NATIVE-PAGE the band of recombinant protein corresponds to ~ 115 kDa. The elusion profile of protein by size exclusion chromatography and DLS also verified the similar results. From these results we conclude that the protein of our interest is an oligomer and it exists in the homotrimeric form in the solution. Since β-mercaptoethanol breaks the disulphide linkage and reduce the protein to its monomeric form, thus the protein with its treatment got linearize and existed in its monomeric form and gave band of ~ 36 kDa but the conversion of trimeric form into monomeric form on SDS-PAGE run even in the absence of a reducing agent suggests the absence of disulphide bonds within the three protomers. Which was further confirmed with mutation of the cysteine residue at position 63 with alanine to see the change in oligomeric structure. Mutation did not give any significant change in the trimeric structure of the protein. Which suggest no role of cys-63 in structure stabilization by disulphide bond formation. Although the presence of disulphide bonds in between cysteine residues of protomers was expected to play role in stabilization of the trimeric structure.

The absorption spectra analysis and TLC concluded that the cofactor FMN is bound to the protein MSMEG_3955. To quantify the number of FMN molecules attached to the protein, apoprotein was obtained by deflavination using different methods i.e. TCA and SDS treatment. Firstly, with the TCA treatment the flavin got detached from the protein and the apoprotein forms insoluble aggregates. We also tried to dissolve the apoprotein using 6 M and 8 M urea but it did not work. Further, the treatment of protein at different SDS concentrations of (0.05–3.0%) showed that deflavination occurred and the apoprotein got degraded at minimum concentration of 0.05% of SDS which showed that the FMN was bound to the protein non-covalently. These results concluded that protein was getting degraded after deflavination and requires FMN for its stability. Similarly, in a study conducted on protein WrbA, an oligomeric flavodoxin-like protein that binds one molecule of flavin mononucleotide (FMN) per monomer was found out to form tetramers, which are more thermoresistant than dimers or monomers, suggesting that multimerization underlies the FMN effect on WrbA thermostability [42]. Furthermore it was also showed a clear shift towards a higher molecular mass of Wild-type dodecin from *S. davaonensis* in the presence of FMN which indicate that the FMN promoted the formation of multimers (probably trimers) [43]. The protein AtHal3 is a homotrimer and three FMN molecules are bound to it, one FMN present between the two adjacent protomers [44]. As FMN bound to the protein at lower temperature [42] and protein MSMEG_3955 also expresses at 25 °C discussed in material and methods. Thus, the existence of trimeric structure of MSMEG_3955 found in the present study could be that there may be three FMN bound to the protein at low temperature, one FMN in between the two adjacent protomers and might provide this protein a homotrimeric stable structure since, no disulphide linkage found within the three protomers to form a stable trimeric structure.

Rv3131 from *Mtb* a close homologue to MSMEG_3955 is considered to be a putative NADPH nitroreductase. The sequence homology suggests that the gene *MSMEG_3955* belongs to nitroreductase family [3]. However, the present study did not show significant nitroreductase activity by purified recombinant protein MSMEG_3955, using nitrofurantoin as substrate and both NADH and NADPH individually as an electron donor. There was no nitroreductase activity compared to the controls (data not shown). It was earlier reported that some flavoenzymes does not show nitroreductase activity [45]. The protein MSMEG_5246 has unknown function and documented as putative nitroreductase, which also does not show nitroreductase activity [19]. The reason for no nitroreductase activity of protein MSMEG_5246 may be assumed for the presence of a lid on the groove of protein’s structure as described by the crystallographic structure as there is no access to the active site. Although, the protein MSMEG_5246 also belong to Acg family [6] and exhibits about 37% identity with MSMEG_3955 and gives top hit model for homologue modelling to generate the hypothetical structure of MSMEG_3955 but do not show FMN reductase activity. However, the cofactor FMN was reduced aerobically by the protein MSMEG_3955 showing FMN reductase activity as discussed in the present study by UV-Visible spectroscopy and ^1^H NMR spectra.

Thus, we are considering presence of FMN reductase activity of the protein MSMEG_3955 is the evolutionary existence of its homotrimeric native form. This structural characterization of the protein MSMEG_3955 is described for the first time in the present study. Since the protein is having FMN reductase activity and because of its existence as trimeric stable conformation, its enzymatic activity can be involved in biochemical pathways which ultimately help in adaptation of bacteria to survive in the dormancy and in the latent infection. The antibiotic treatment against TB requires a number of drugs depending upon its occurrence such as multiple drug resistant (MDR) and extremely drug resistant (XDR). These drugs work against different components of TB. Elucidating a distinct activity of *Mtb* proteins can help in better immune surveillance and therefore can be used in the future control of TB.

## Conclusion

Our study conclude, the MSMEG 3955 protein is a flavoprotein that is bound by the FMN. In its natural state, it exists as a homotrimer. There are no disulphide linkages between the three protomers of homotrimer MSMEG 3955. This protein is a NADPH-dependent oxidoreductase that uses FMN as a cofactor.

## Supplementary Information

**Additional file 1 **: **Figure S1**. Sequence Alignment of *MSMEG_3955* from *Mycobacterium smegmatis* MC2–155 with *Rv3131* from *Mycobacterium tuberculosis* H37Rv showing Identities: 202/323(63%), Positives: 249/323(77%), Gaps: 0/323(0%). **Figure S2**. The 1H NMR spectra of FMN oxidised form recorded at 500 MHz, pH -7.6. **Figure S3** The 1H NMR spectra of FMN in oxidized (red) and reduced (blue) form recorded at 500 MHz, pH -7.6. **Figure S4**. Validation of generated monomeric MSMEG_3955. a) PROCHECK gave results as 89.9% in most favoured regions, 8.3% in additional allowed regions, 1.4% in generously allowed regions and 0.4% in disallowed regions. b) Verify 3D gave 96.68% showing the generated trimeric protein model of good quality. c) ERRAT gave overall quality factor 90.7 expressed as the percentage of the protein for which the calculated error value falls below 95% rejection limit. **Figure S5.** Absorbance-time curve for the reaction of Protein + NADPH + FMN.

## Data Availability

The dataset used in the current study was submitted to NCBI (GenBank) and the accession number is MZ198152.

## Abbreviations

Acg: Acr co-regulated gene
BLAST: Basic Local Alignment Search Tool
BSA: Bovine serum albumin
CD: Circular Dichroism
cDNA: Complementary DNA
CTAB: Hexadecyltrimethyl ammonium bromide
DLS: Dynamic Light Scattering
DosR: Dormancy survival regulator
FMN: Flavin mononucleotide
GPC: Gel permeation chromatography
IPTG: Isopropyl β-D-1-thiogalactopyranoside
LB: Luria bertani
MDR: Multiple drug resistant
NADH: Nicotinamide adenine dinucleotide (Reduced form)
NADPH: Nicotinamide adenine dinucleotide phosphate (Reduced form)
NCBI: National Center for Biotechnology Information
Ni-NTA: Nickel-Nitrilotriacetic Acid
NMR: Nuclear magnetic resonance
OADC: Oleic acid dextrose catalase
PAGE: Polyacrylamide Gel Electrophoresis
PCR: Polymerase Chain Reaction
PDB: Protein Data Bank
PMSF: Phenylmethylsulfonyl fluoride
SDM: Site directed mutagenesis
SDS: Sodium dodecyl sulfate
SEC: Size exclusion chromatography
TCA: Trichloroacetic acid
TLC: Thin layer chromatography
UV: Ultraviolet
XDR: Extremely drug resistant
