# Supplementary material for: MSMEG_3955 from Mycobacterium smegmatis is a FMN bounded homotrimeric NAD(P)H:Flavin mononucleotide (FMN) oxidoreductase
Source: BMC Microbiol. 2021 Nov 19;21:319. doi: 10.1186/s12866-021-02330-y (PMC8605562; doi:10.1186/s12866-021-02330-y)
Supplement: Supplementary file 1 — Additional file 1 : Figure S1. Sequence Alignment of MSMEG_3955 from Mycobacterium smegmatis MC2–155 with Rv3131 from Mycobacterium tuberculosis H37Rv showing Identities: 202/323(63%), Positives: 249/323(77%), Gaps: 0/323(0%). Figure S2. The 1H NMR spectra of FMN oxidised form recorded at 500 MHz, pH -7.6. Figure S3 The 1H NMR spectra of FMN in oxidized (red) and reduced (blue) form recorded at 500 MHz, pH -7.6. Figure S4. Validation of generated monomeric MSMEG_3955. a) PROCHECK gave results as 89.9% in most favoured regions, 8.3% in additional allowed regions, 1.4% in generously allowed regions and 0.4% in disallowed regions. b) Verify 3D gave 96.68% showing the generated trimeric protein model of good quality. c) ERRAT gave overall quality factor 90.7 expressed as the percentage of the protein for which the calculated error value falls below 95% rejection limit. Figure S5. Absorbance-time curve for the reaction of Protein + NADPH + FMN. [file 12866_2021_2330_MOESM1_ESM.docx]

MSMEG_3955 from *Mycobacterium smegmatis* is a FMN bounded homotrimeric NAD(P)H:Flavin mononucleotide (FMN) oxidoreductase.

Neha Khosla^1^, Seema Madhumal Thayil^1^, Rajinder Kaur^2^ and Anup Kumar Kesavan^1^*

^1^Department of Molecular Biology and Biochemistry*,* Guru Nanak Dev University, Amritsar, 143005, Punjab, India.

^2^Department of Botanical and Environmental Sciences, Guru Nanak Dev University, Amritsar, 143005, Punjab, India.

# *Corresponding author

# Dr. Anup Kumar Kesavan

Department of Molecular Biology and Biochemistry

Guru Nanak Dev University

Amritsar, Punjab, 143005, India

Ph.: +91-8968654186

Email: [akesav@gmail.com](mailto:akesav@gmail.com)

**Supplementary Information**

MSMEG_3955 1 MNTHFPDTETIHAALALAMRAPSIHNSQPWQWRVGERSVHLYADLDRHLTSTDPDSRDLL 60

MNTHFPD ET+ L LA+RAPSIHN+QPW+WRV S+ L++ D L STDPD R+L+

Rv3131 1 MNTHFPDAETVRTVLTLAVRAPSIHNTQPWRWRVCPTSLELFSRPDMQLRSTDPDGRELI 60

MSMEG_3955 61 LSCGAALHHCVIAFAALGWYASVHRLPNPAEPEHLASIELRRQTPTDLDIALAAAIPRRR 120

LSCG ALHHCV+A A+LGW A V+R P+P + HLA+I ++ P D+ALAAAIPRRR

Rv3131 61 LSCGVALHHCVVALASLGWQAKVNRFPDPKDRCHLATIGVQPLVPDQADVALAAAIPRRR 120

MSMEG_3955 121 TDRRHYSAWPVSHSDIAMMGARAARAGVMLRRVESLSRLQDIVAESIARHAADDGYLREL 180

TDRR YS WPV DIA+M ARAAR GVMLR+V +L R++ IVA+++ H D+ YLREL

Rv3131 121 TDRRAYSCW0PVPGGDIALMAARAARGGVMLRQVSALDRMKAIVAQAVLDHVTDEEYLREL 180

MSMEG_3955 181 TAWSGKYASTAGVPARSAPKPEPGAPLSSRMFAGAALKQPDTGAGADEGAVVLALGTADD 240

T WSG+Y S AGVPAR+ P +P AP+ R+FAG L QP AD+GA +LALGT D

Rv3131 181 TIWSGRYGSVAGVPARNEPPSDPSAPIPGRLFAGPGLSQPSDVLPADDGAAILALGTETD 240

MSMEG_3955 241 TRMSRLRAGEATSLILLTATAMGLATCPVTEPLEISETRDAVQAEVFGASAFPQMMLRVG 300

R++RLRAGEA S++LLTATAMGLA CP+TEPLEI++TRDAV+AEVFGA +PQM+LRVG

Rv3131 241 DRLARLRAGEAASIVLLTATAMGLACCPITEPLEIAKTRDAVRAEVFGAGGYPQMLLRVG 300

MSMEG_3955 301 WAPVNADPLPPTPRRHVADAVRW 323

WAP+NADPLPPTPRR ++ V W

Rv3131 301 WAPINADPLPPTPRRELSQVVEW 323

**Fig. S1:** Sequence Alignment of *MSMEG_3955* from Mycobacterium smegmatis MC2-155 with *Rv3131* from Mycobacterium tuberculosis H37Rv showing Identities: 202/323(63%), Positives: 249/323(77%), Gaps: 0/323(0%).


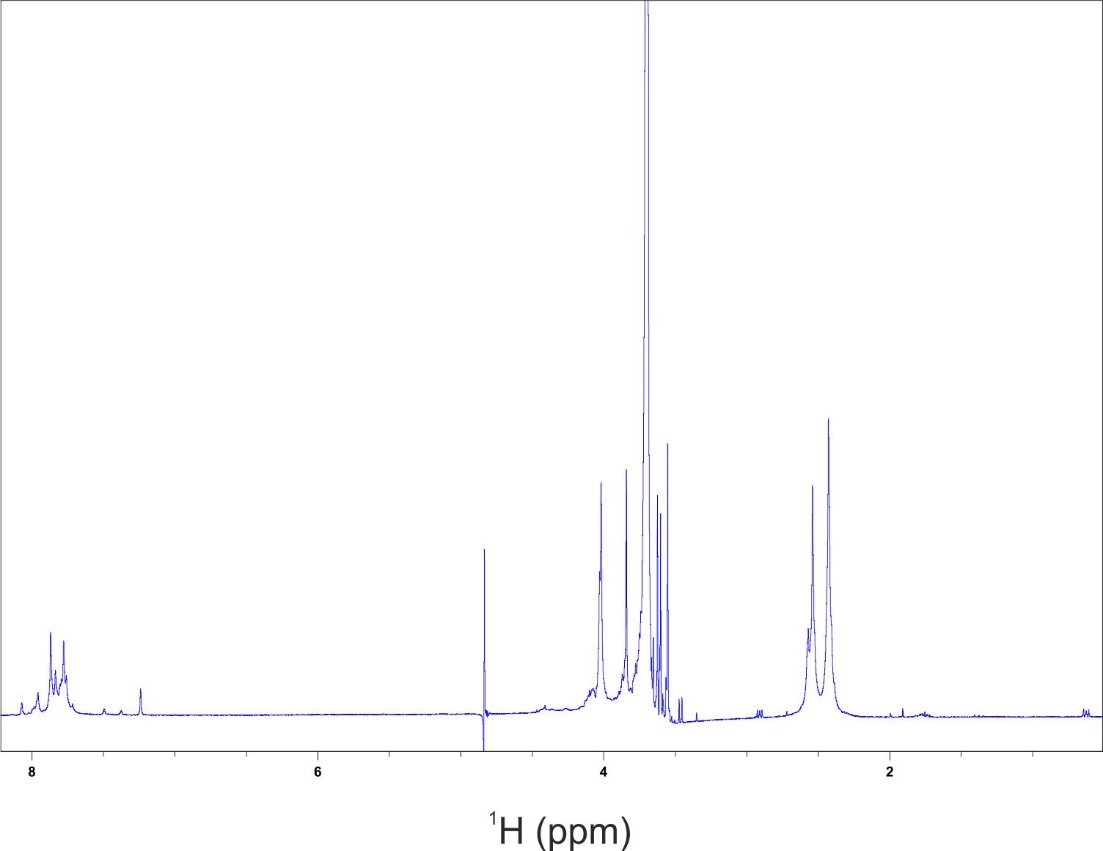


**Fig. S2:** The ^1^H NMR spectra of FMN oxidised form recorded at 500MHz, pH-7.6.

**\**


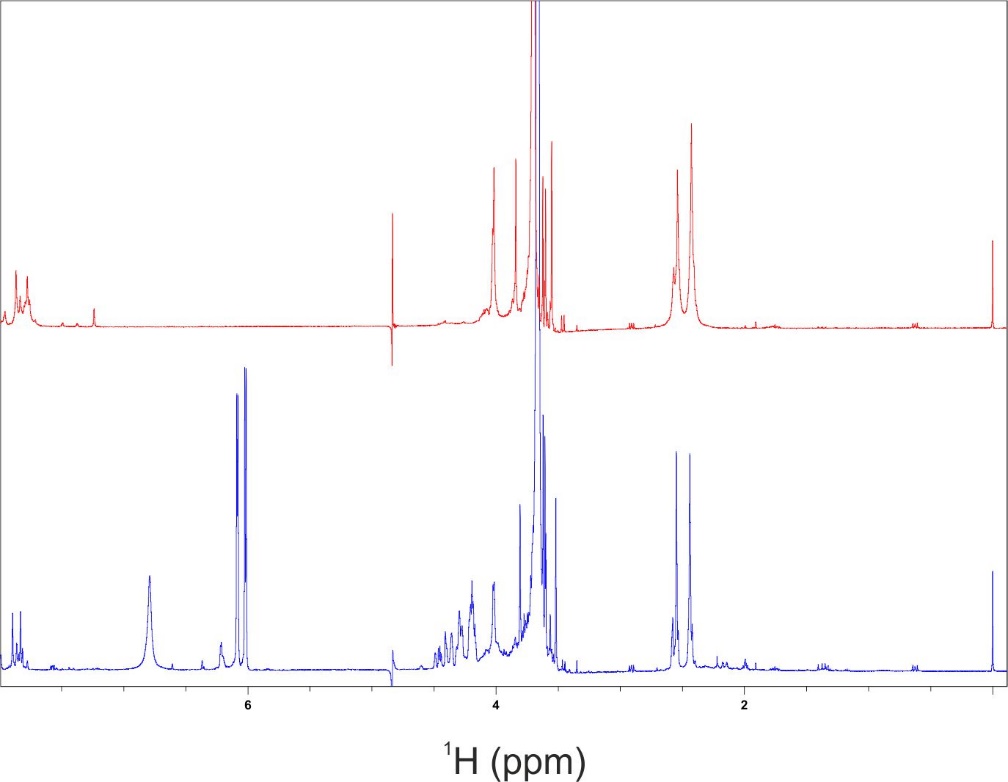


**Fig. S3:** The ^1^H NMR spectra of FMN in oxidized (red) and reduced (blue) form recorded at 500MHz, pH-7.6.

**a)**


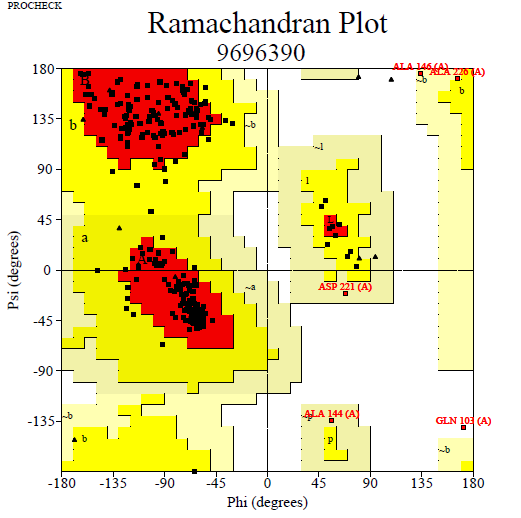


**
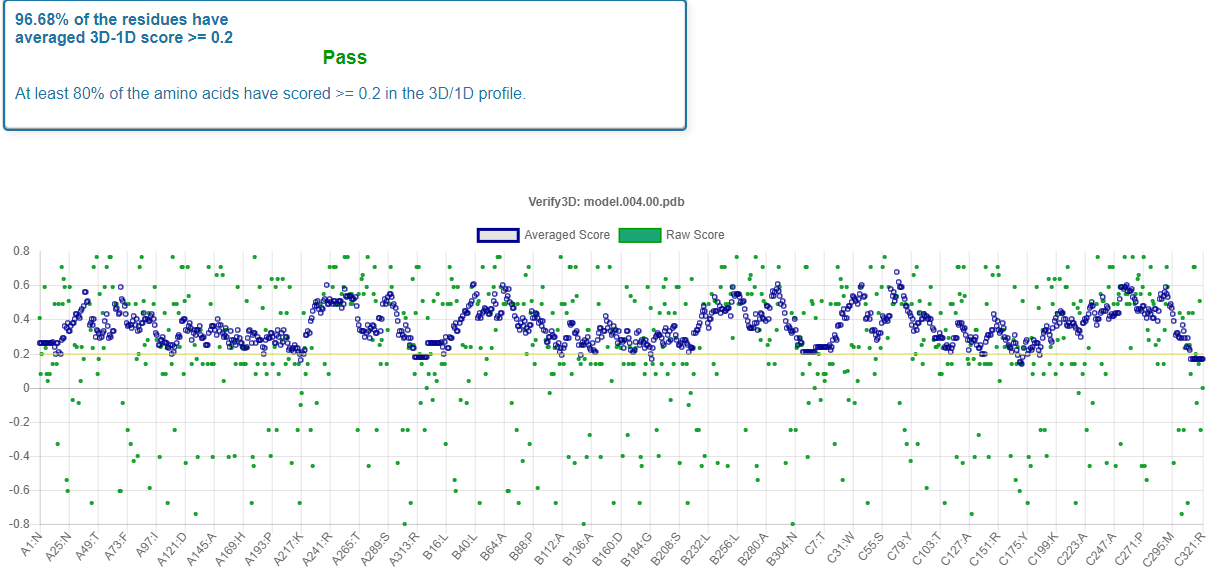
**

**b)**

**
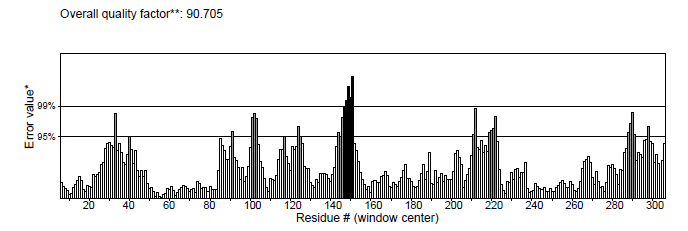
**

**c)**

**Fig S4. Validation of generated monomeric MSMEG_3955. a)** PROCHECK gave results as 89.9% in most favoured regions, 8.3% in additional allowed regions, 1.4% in generously allowed regions and 0.4% in disallowed regions. **b)** Verify 3D gave 96.68% showing the generated trimeric protein model of good quality. **c)** ERRAT gave overall quality factor 90.7 expressed as the percentage of the protein for which the calculated error value falls below 95% rejection limit.


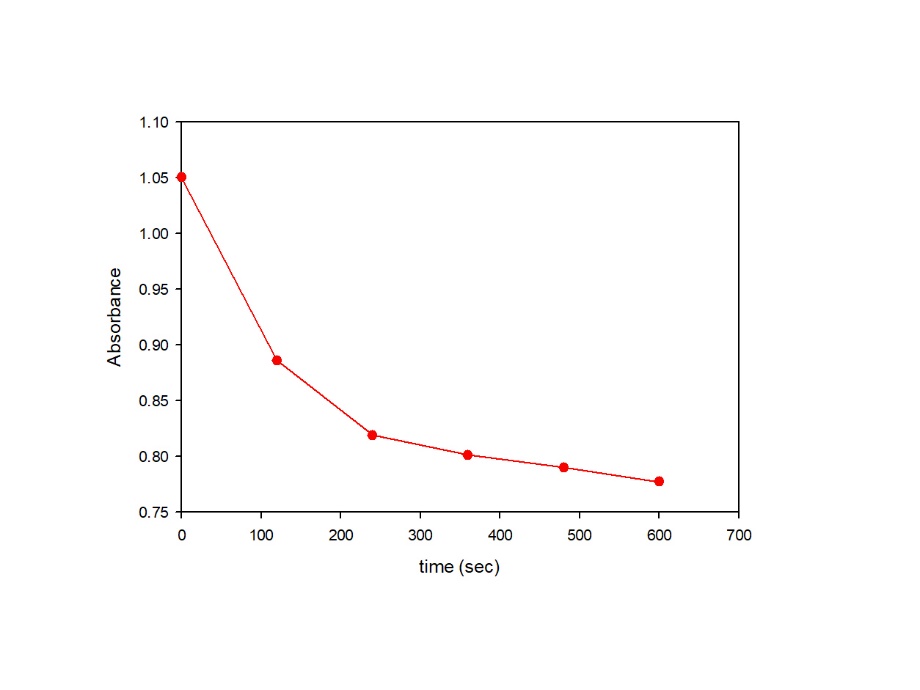


**Fig S5:** Absorbance-time curve for the reaction of Protein + NADPH + FMN.
